# Treatment strategies and prognosis of superficial laryngo-pharyngeal cancer: a literature review

**DOI:** 10.1007/s10147-025-02781-7

**Published:** 2025-07-07

**Authors:** Kazuchika Ohno, Takahiro Asakage

**Affiliations:** Department of Head and Neck Surgery, Institute of Science Tokyo Hospital, 1-5-45, Yushima, Bunkyo-Ku, Tokyo, 113-8519 Japan

**Keywords:** Superficial laryngo-pharyngeal cancer, Organ preservation, Transoral surgery, Lymph node metastasis, Metachronous cancers

## Abstract

Superficial laryngo-pharyngeal cancer (SLPC) is defined as that stage of the cancer in which “cancer cells are confined to the subepithelial layer, without invasion of the muscularis propria, with or without lymph node metastasis.” With the advances in endoscopic technologies and observation methods, numerous cases of SLPC have been reported in recent years. Less invasive oral resection methods, enabling organ preservation, have also been developed for the treatment of SLPC. However, it should be noted that the diagnosis of SLPC is based on the tumor thickness, which cannot be addressed by the TNM classification. Furthermore, although SLPC is generally associated with a good prognosis, a certain proportion of patients develop lymph node metastasis and/or multiple metachronous cancers, both of which may be expected to have an adverse impact on the prognosis. In addition, sufficient evidence has not accumulated for optimal post-treatment surveillance and factors affecting the risk of lymph node metastasis, and further investigation is required. In this review, we describe the epidemiology, general characteristics, diagnosis, treatment, and prognosis of SLPC.

## Introduction

**Fig. 1:**
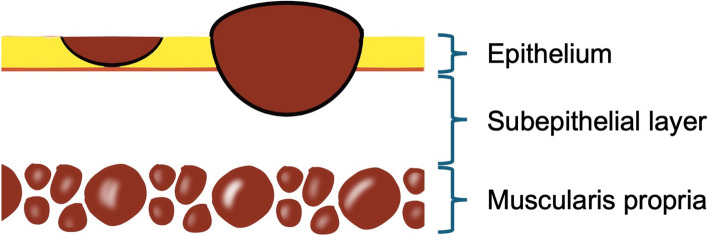
Schematic view of the pathological classification of SLPC, modified from reference 6

In the past, head and neck cancers, especially oropharyngeal and hypopharyngeal cancers, were often detected at an advanced stage and were associated with a poor prognosis [1, 2]. However, since the beginning of 2000, with the advancements in technologies such as narrow-band imaging (NBI) and magnifying endoscopy, the accuracy of endoscopic diagnosis has improved significantly, and many cases of head and neck cancer are now detected at an early stage [3–5]. Therefore, in 2018, the Japan Society for Head and Neck Cancer published the Guideline for the Management of Superficial Cancer of the Head and Neck, establishing criteria for the diagnosis of superficial laryngo-pharyngeal cancer (SLPC). According to this guideline, SLPC is defined as “cancer cells are confined to the subepithelial layer, without invasion of the muscularis propria, with or without lymph node metastasis” [6] (Fig. 1). However, SLPC is defined based on the tumor thickness, as discussed below, which cannot be addressed by the current TNM classification, which is based on the tumor size. Furthermore, it is important to note that the presence/absence of lymph node metastasis is not defined, and even superficial cancer can be in various stages. Since SLPC is a new disease concept, caution must be exercised in the diagnosis and treatment of this disease. In this review, we discuss the epidemiology, general characteristics, diagnosis, treatment, and prognosis of SLPC.

## Epidemiology and general characteristics

The reported odds ratios for the development of hypopharyngeal and esophageal cancer in heavy drinkers (≥ 1.5 drinks/day), heavy smokers (≥ 30 packs-years), and heavy drinkers with a heavy smoking habit are 8.2, 3.9, and 29.9, respectively [7]. The higher risk of development of head and neck cancer and esophageal cancer associated with heavy alcohol consumption is considered as being attributable to accumulation of acetaldehyde, the main metabolite of alcohol. In particular, people with deficiency of aldehyde dehydrogenase 2 (ALDH2) (the enzyme that breaks down acetaldehyde) who consume alcohol show accumulation of acetaldehyde in their bodies and an increased risk of development of head and neck cancer and esophageal cancer [8]. In addition, while studies have suggested that besides the drinking habit and smoking habit, insufficient intake of green and yellow vegetables and polymorphisms of *ALDH2* and *ADH1B* are also risk factors for carcinogenesis [9], the major risk factors for the development of SLPC remain drinking and smoking [10].

The proportion of cases of SLPC in the overall head and neck cancer population is still unknown due to the lack of big data. However, with advances in endoscopic technologies, according to the head and neck cancer registry of Japan, the rate of diagnosis of Tis, which is largely considered as superficial cancer, increased from 6 cases in 2011 to 52 cases 2020 for oropharyngeal cancer, from 31 to 143 cases during the same period for hypopharyngeal cancer, and from 2 to 10 cases during the same period for supraglottic cancer [11, 12]; the rate of diagnosis is expected to increase further in the future.

The natural history of SLPC has not yet been clearly elucidated. Nakamura et al. reported that SLPC, if left untreated, showed a rapid increase in tumor size, although no deaths have been attributed to progression of SLPC. However, the appearance of endoscopic findings suggestive of submucosal invasion within one year of the initial diagnosis in a case diagnosed as having carcinoma in situ by endoscopy has led to the recommendation of early initiation of treatment [13]. Several studies have reported that carcinoma in situ is not associated with a risk of lymph node metastasis [14, 15], so that care should be taken not to miss the timing of treatment.

## Diagnosis

**Fig. 2:**
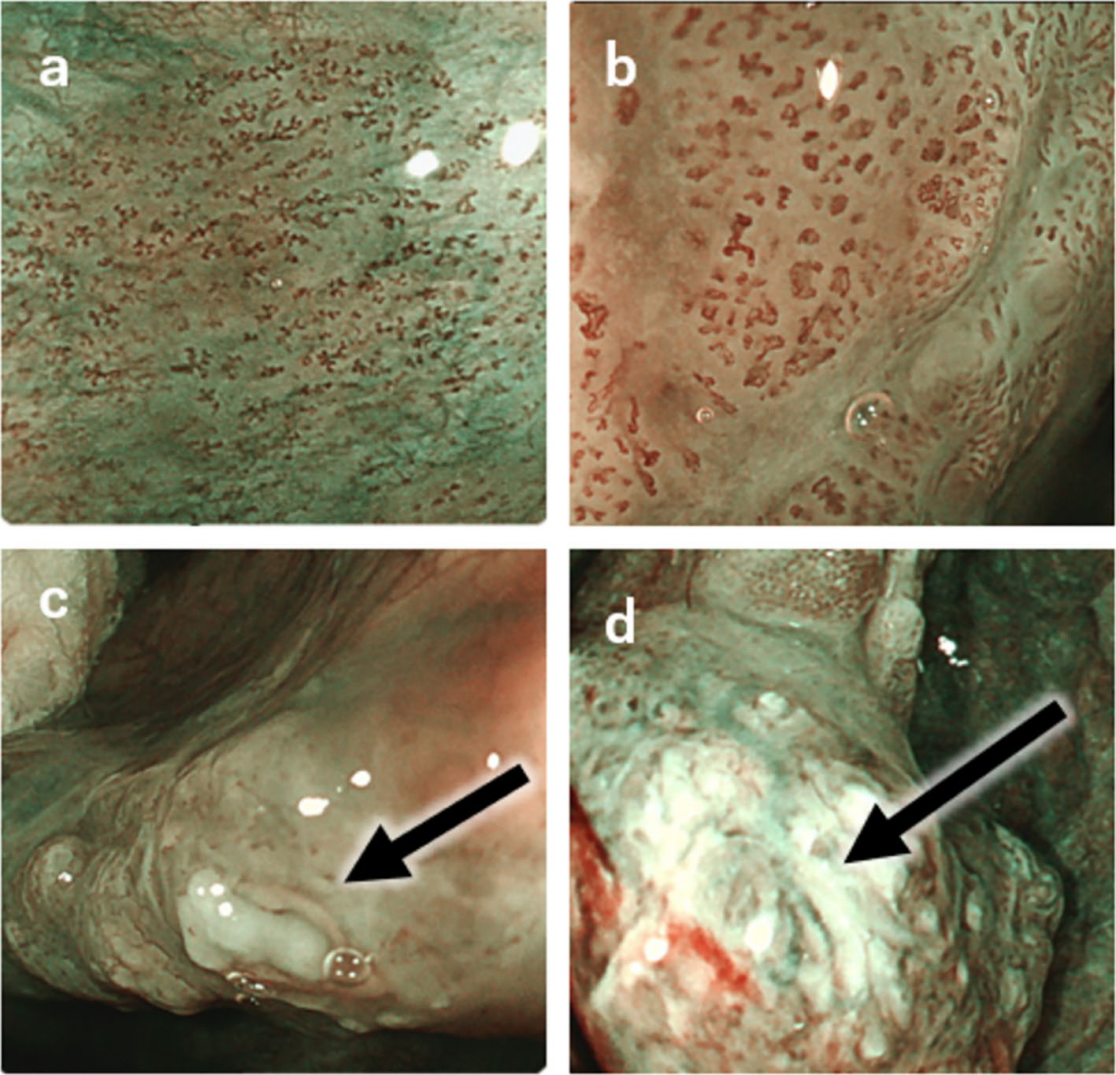
IPCLs. **a** JES-Type A (inflammation): There is no evidence of dilatation or tortuosity of the vessels, no irregularity in caliber or shape. **b** JES-Type B1: The loop structure of the vessels is preserved, but some dilatation and tortuosity are observed. Uneven caliber and shape are also noted. **c** JES-Type B2: Abnormal vessels with no loop structure. **d** JES-Type B3: Abnormal vessels of cyanotic tone with more than 3 times the caliber of B2 vessels

Endoscopic examination is primarily used for the diagnosis of SLPC, but the advent of narrow-band imaging (NBI) has enabled early detection of superficial cancer, which is more difficult to detect by conventional endoscopy [16, 17]. However, the NBI endoscopes used in the field of otorhinolaryngology lack an optical zoom function, which makes it difficult to observe the characteristic vascular patterns (intra-papillary capillary loops; IPCLs) (Fig. 2), even though brownish areas can be visualized. Therefore, in many cases, an upper gastrointestinal magnifying endoscope with NBI is used to detect early-stage squamous cell carcinoma of the head and neck, which is difficult to detect by conventional endoscopy, as it allows delineation of IPCLs and the boundary between the lesion and normal tissue at a high probability [18].

**Fig. 3:**
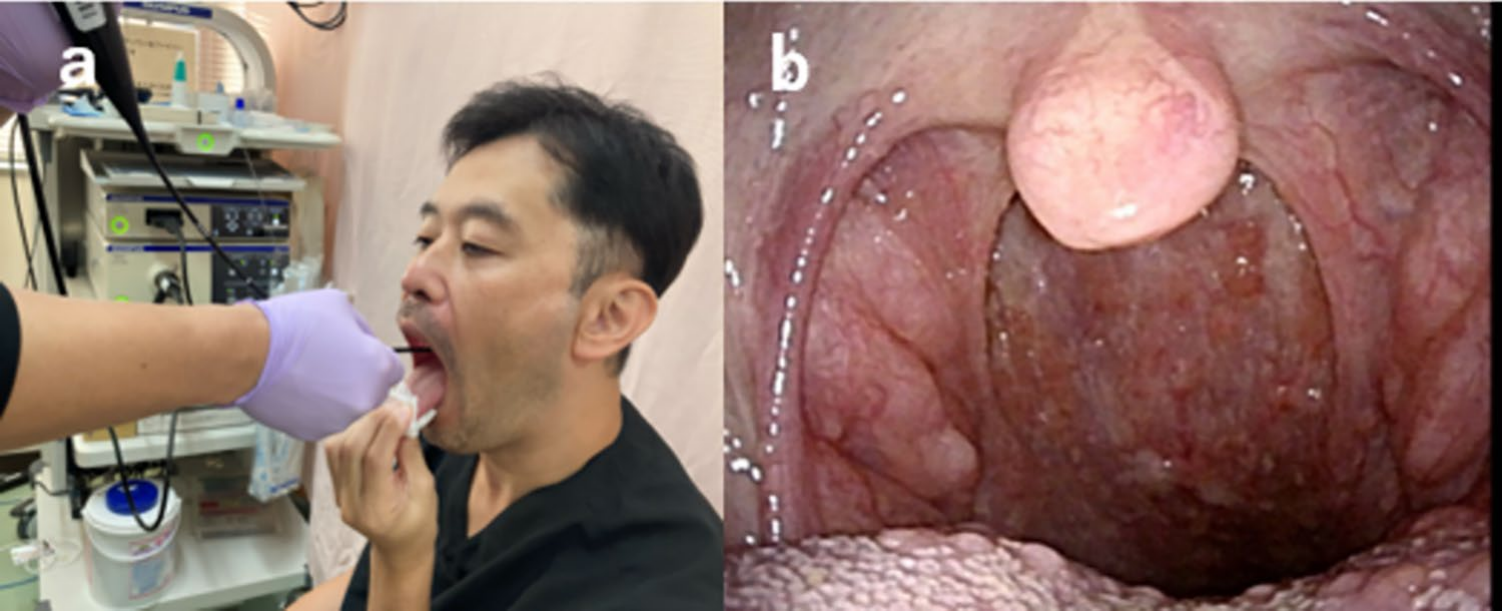
Transoral observation method. **a** The patient pulls the tongue forward and the pharynx is observed transorally. **b** Endoscopic view of oropharynx in transoral observation method

**Fig. 4:**
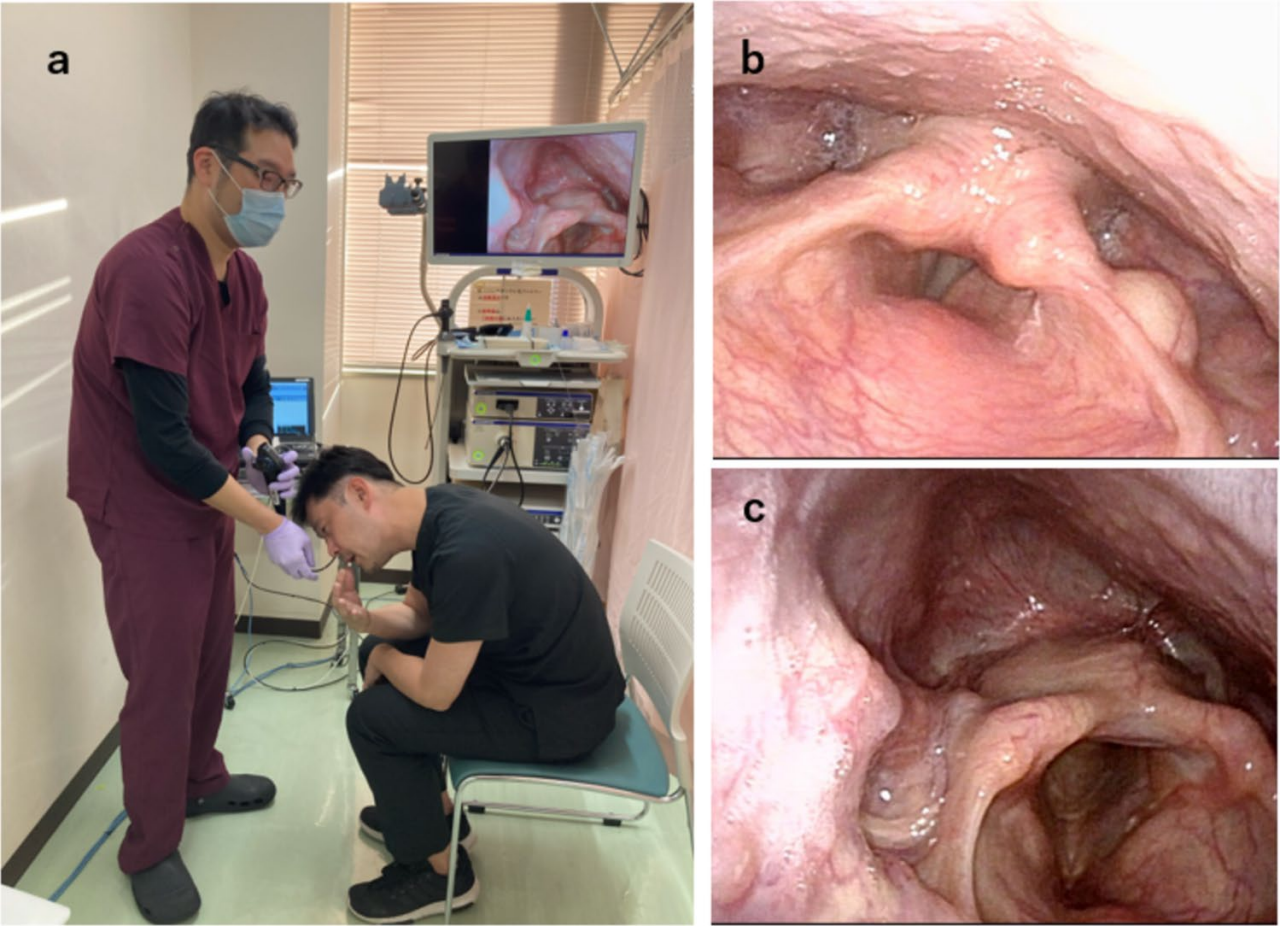
The modified Killian’s method. **a** The patient assumes a forward-bending position while pulling the chin. **b** Endoscopic view of hypopharynx in normal head position. **c** Endoscopic view of hypopharynx in modified killian’s method

**Fig. 5:**
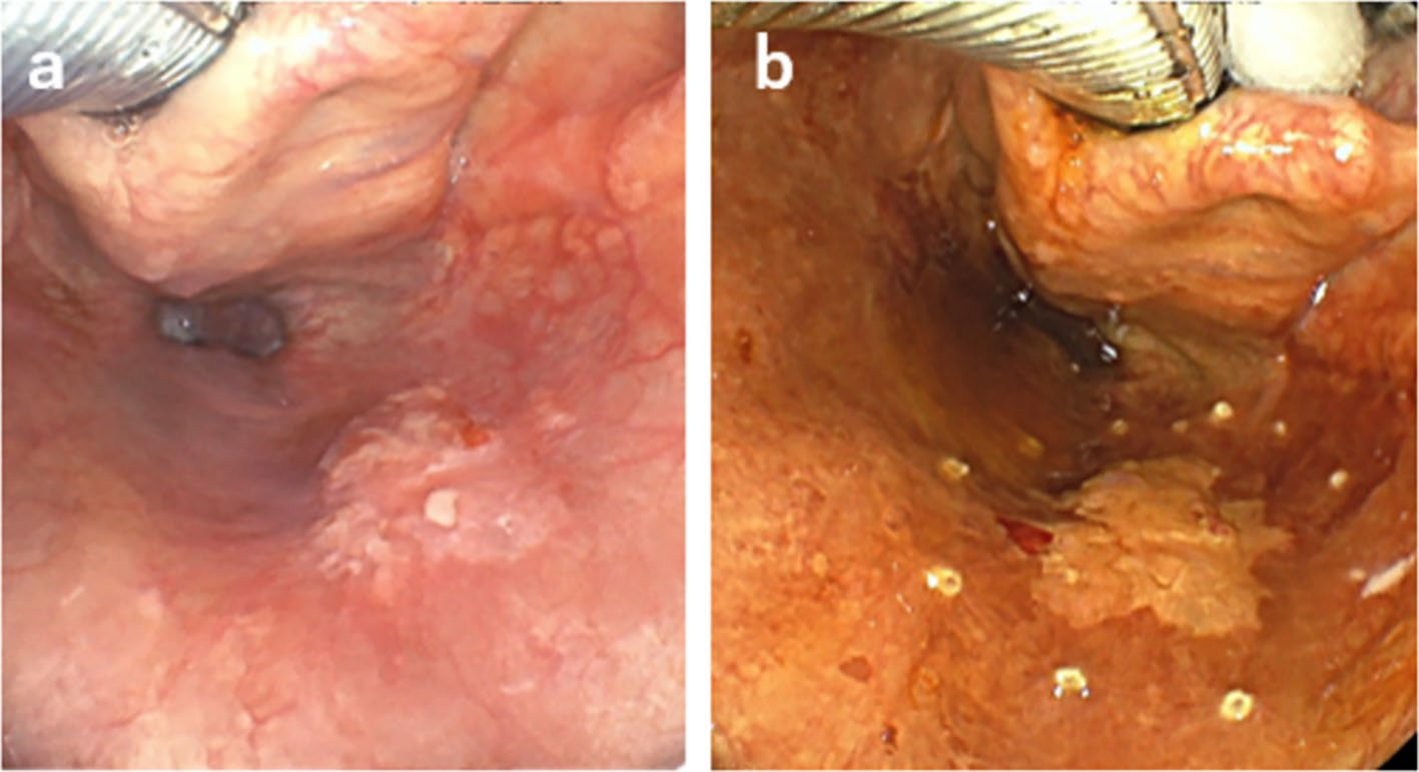
**a** Posterior wall lesion of the hypopharynx observed in normal light. **b** Lugol dye solution shows the tumor area as an unstained lesion

**Fig. 6:**
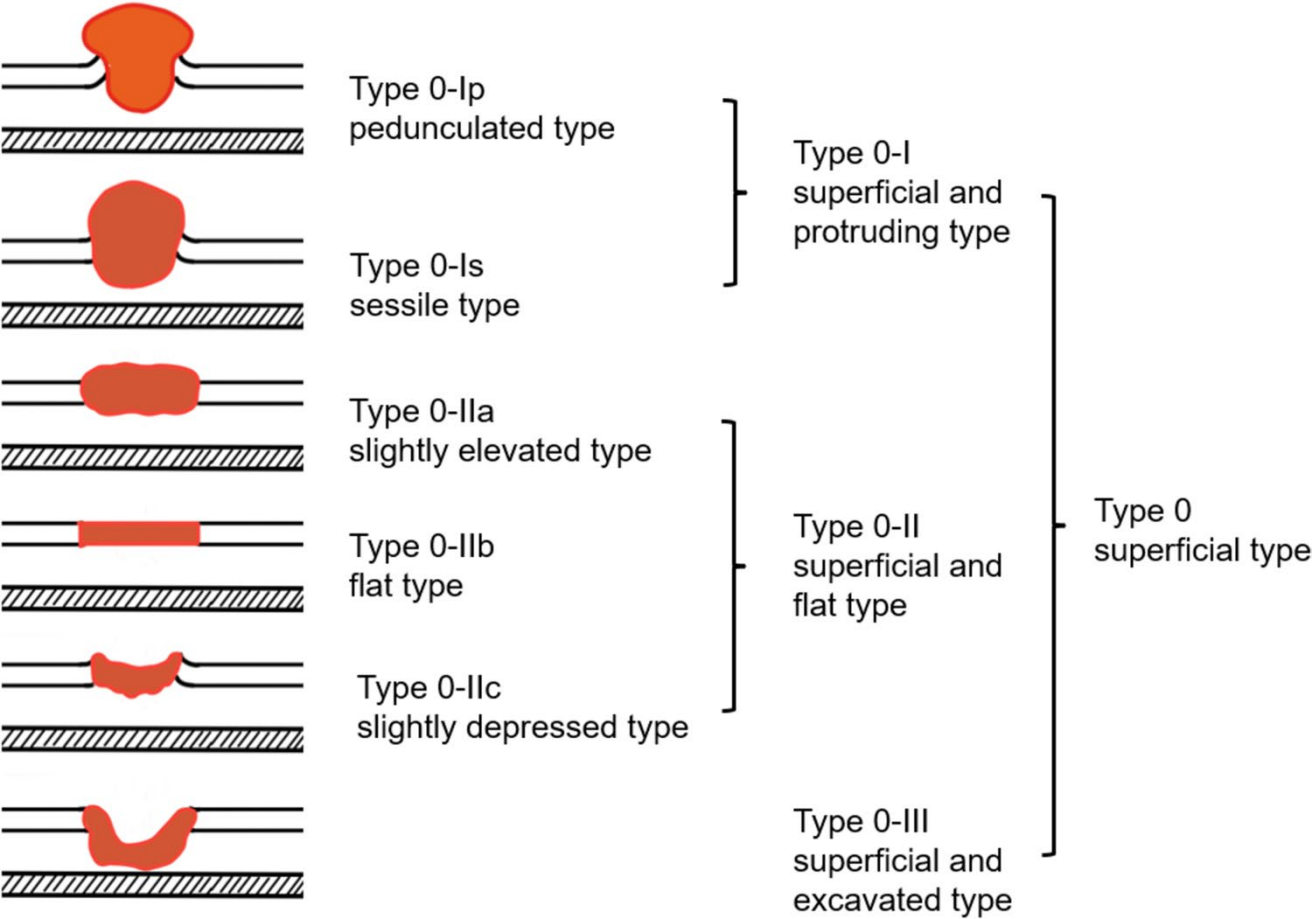
Schematic view of the macroscopic classification of SLPC, modified from reference 6

In addition to these endoscopic techniques, more detailed observation is possible by using the “Transoral observation method” for observation of the oropharynx (Fig. 3), and the “Modified Killian’s method” for observation of the hypopharynx [19, 20] (Fig. 4). As for lesions in the esophageal region, the Lugol dye solution method is also useful for defining the extent of the lesion. Lugol dye solution makes it easier to identify the extent of the lesion, because the normal mucosa stains brown, whereas cancerous lesions appear unstained and white (Fig. 5). In the pharyngeal and laryngeal regions, however, Lugol dye can only be used under general anesthesia due to its highly irritating effects. Fluorescence imaging using γ-glutamyl hydroxymethyl rhodamine green (gGlu-HMRG), a fluorescent agent activated by a cell surface enzyme called γ-glutamyltranspeptidase (GGT), which is overexpressed in some cancers, may be useful for early detection of head and neck squamous cell carcinoma [21, 22]. While the Lugol dye solution method and fluorescence imaging are helpful to assess the tumor extent, it is equally important to accurately assess the tumor depth. For measurement of the lesion depth, macroscopic classification (Fig. 6) and the magnifying endoscopic classification of the Japan Esophageal Society to determine the invasion depth in the diagnosis of superficial esophageal cancer are useful [23, 24], and if the lesion is endoscopically diagnosed as 0–I or 0–II, there is a low probability of muscular invasion [25]. For a more precise diagnosis of the depth, oral ultrasound endoscopy is also useful [26, 27]. Based on the results of these examinations, the diagnosis of SLPC is confirmed and a treatment plan is determined.

## Treatment

Several transoral approaches have been developed and reported for the treatment of SLPC, that allow organ preservation and do not cause mucositis or xerostomia, which are common complications of chemoradiation therapy. These approaches are collectively referred to as transoral surgery (TOS). The following disease types are currently considered as indications for TOS:

- Indications
- No lymph node metastasis on preoperative examination
- Endoscopic diagnosis of carcinoma in situ or subepithelial invasive carcinoma

The advantages of TOS, besides a minimally invasive approach that does not involve skin incisions, include reduced tissue detachment, minimal impairment of the speech and swallowing muscles, limited bleeding, minimal damage of major neurovascular structures, and limited damage of normal tissues. In this overview, we will discuss typical TOS techniques.

1. Transoral laser microsurgery (TLM).

As a pioneer of TOS, Steiner et al. reported TLM using a CO_2_ laser under a surgical microscope in the 1980 s to resect hypopharyngeal cancer. They reported favorable results, with a 5-year disease-free survival rate of 95% in Stage I and II patients, and of 69% in Stage III and IV patients, and a 5-year overall survival rate (OS) of 71% in Stage I and II patients, and of 47% in Stage III and IV patients [28, 29]. However, due to the need for advanced techniques such as tumor resection and margin determination, as well as extensive experience, it did not come to be widely adopted.

2. Endoscopic mucosal resection (EMR), Endoscopic submucosal dissection (ESD)

**Fig. 7:**
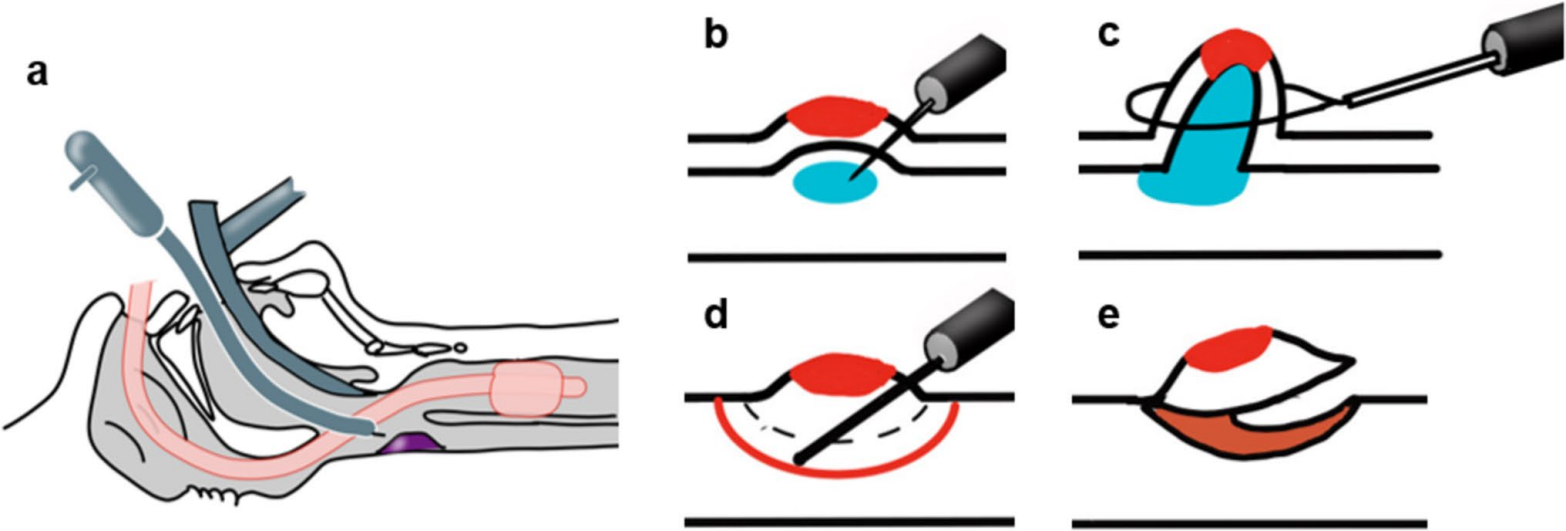
**a** Schematic view of EMR and ESD. **b**, **c** Schematic view of EMR. Saline is injected into the subepithelial propria and the tumor is resected using a snare. **d**, **e** Schematic view of ESD. Saline is injected into the subepithelial propria. After marking with an electrocautery scalpel, resect outside of it

Since gastrointestinal endoscopy is useful for the diagnosis of SLPC, as mentioned above, Muto et al. applied EMR, which is an established treatment technique for superficial cancer of the esophagus, for treating SLPC [16]. However, EMR involves resecting the mucosa by suctioning it into a cap connected to the endoscope and has the disadvantage that accurate resection lines cannot be confirmed. To overcome this disadvantage, Shimizu et al. performed ESD, in which a needle knife is used to resect the mucosa (Fig 7), which results in a larger *en-bloc* resection specimen as compared to EMR. With appropriate selection of cases, both techniques have yielded good results [30].

3. Transoral videolaryngoscopic surgery (TOVS)

**Fig. 8:**
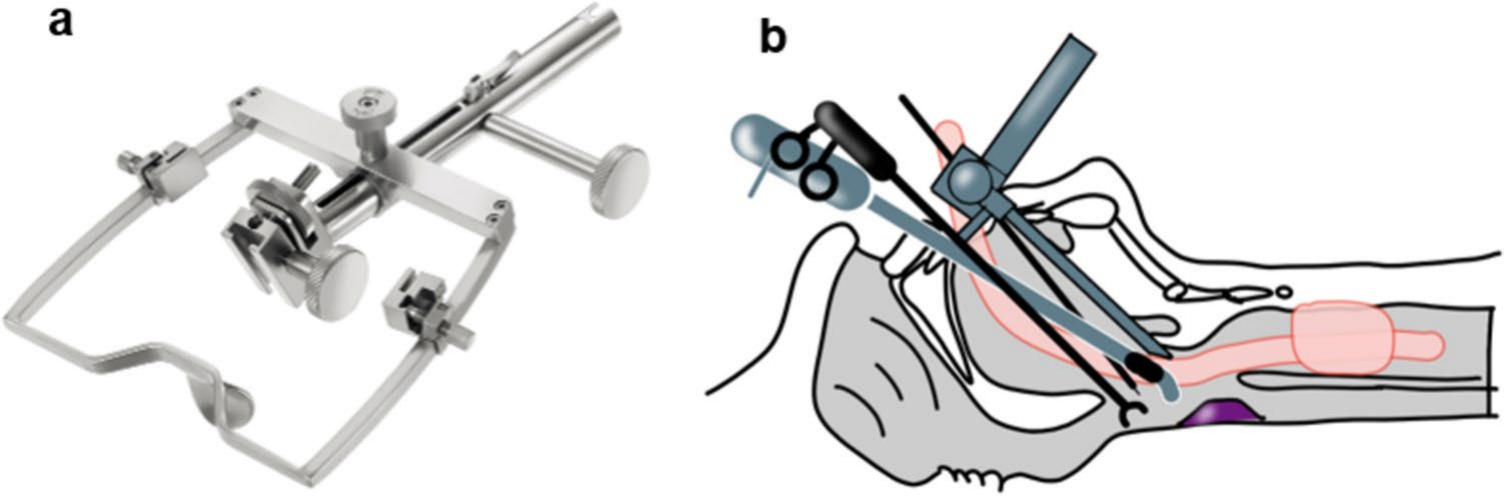
**a** FK larygopharyngoscope (Courtesy of cOlympus Marketing, Inc.) **b** Schematic view of TOVS. The hypopharynx is widened with a FK laryngopharyngoscope and operated on with straight devices

TOVS, which allows the disadvantages of TLM such as a poor field of view under the microscope to be overcome, and allows segmental resection through use of the Weerda distending laryngoscope (Karl Storz, Tuttlingen, Germany), FK retractor (Courtesy of cOlympus Marketing,Inc, Tokyo), and rigid videolaryngoscope, was developed by Shiotani et al. [31, 32] (Fig 8). Indications for TOVS include T1, T2, and selected cases of T3 supraglottic and hypopharyngeal cancers without deep infiltration of either the pharyngeal muscle or thyroid cartilage. Other advantages of TOVS are that the surgeon can use both hands, the instruments used are straightforward and are therefore easy for the surgeon to imagine, and furthermore, the surgeon can directly perceive tactile sensations through the forceps and electrocautery [33].

4. Endoscopic laryngo-pharyngeal surgery (ELPS)

**Fig. 9:**
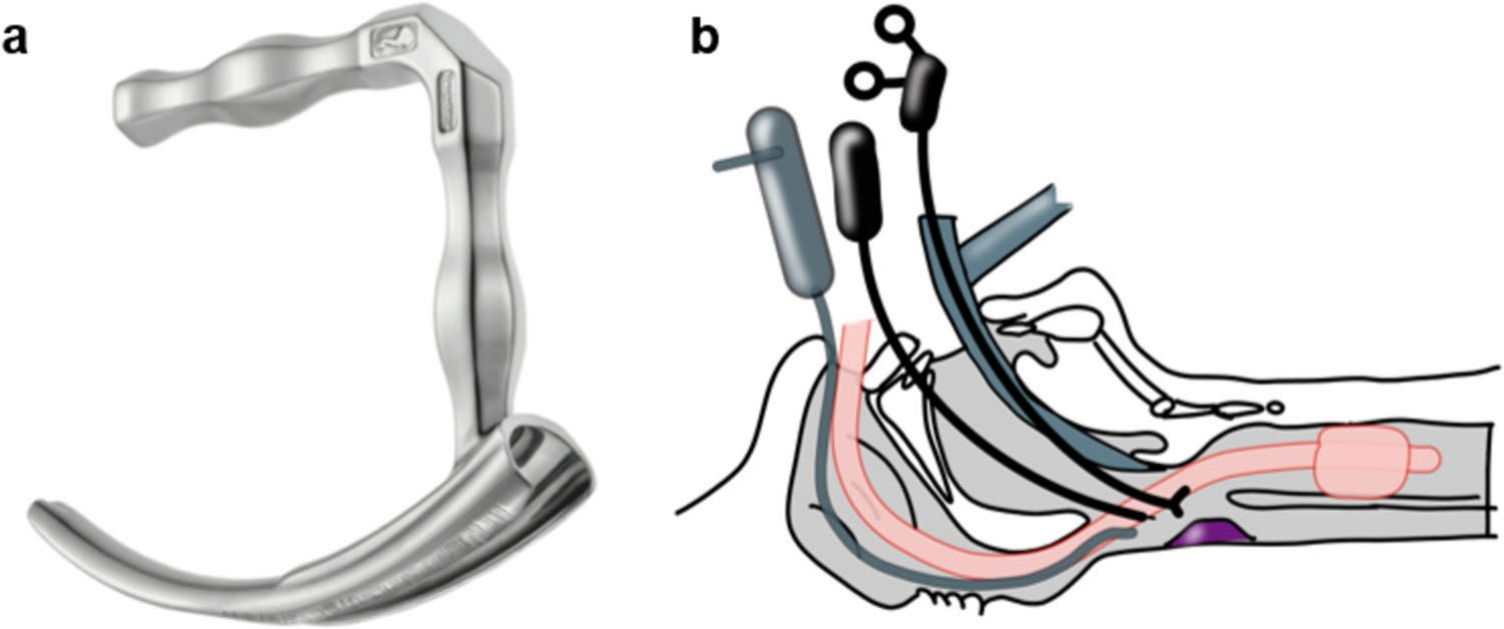
**a** Curved laryngoscope (Reprinted from Nagasima Medical Instruments Company, LTD website. https://www.nagashima-medical.co.jp/wp-content/uploads/2022/10/T-27.pdf. **b** Schematic view of ELPS. The hypopharynx is widened with a curved laryngoscope and operated on with curved devices

**Fig. 10:**
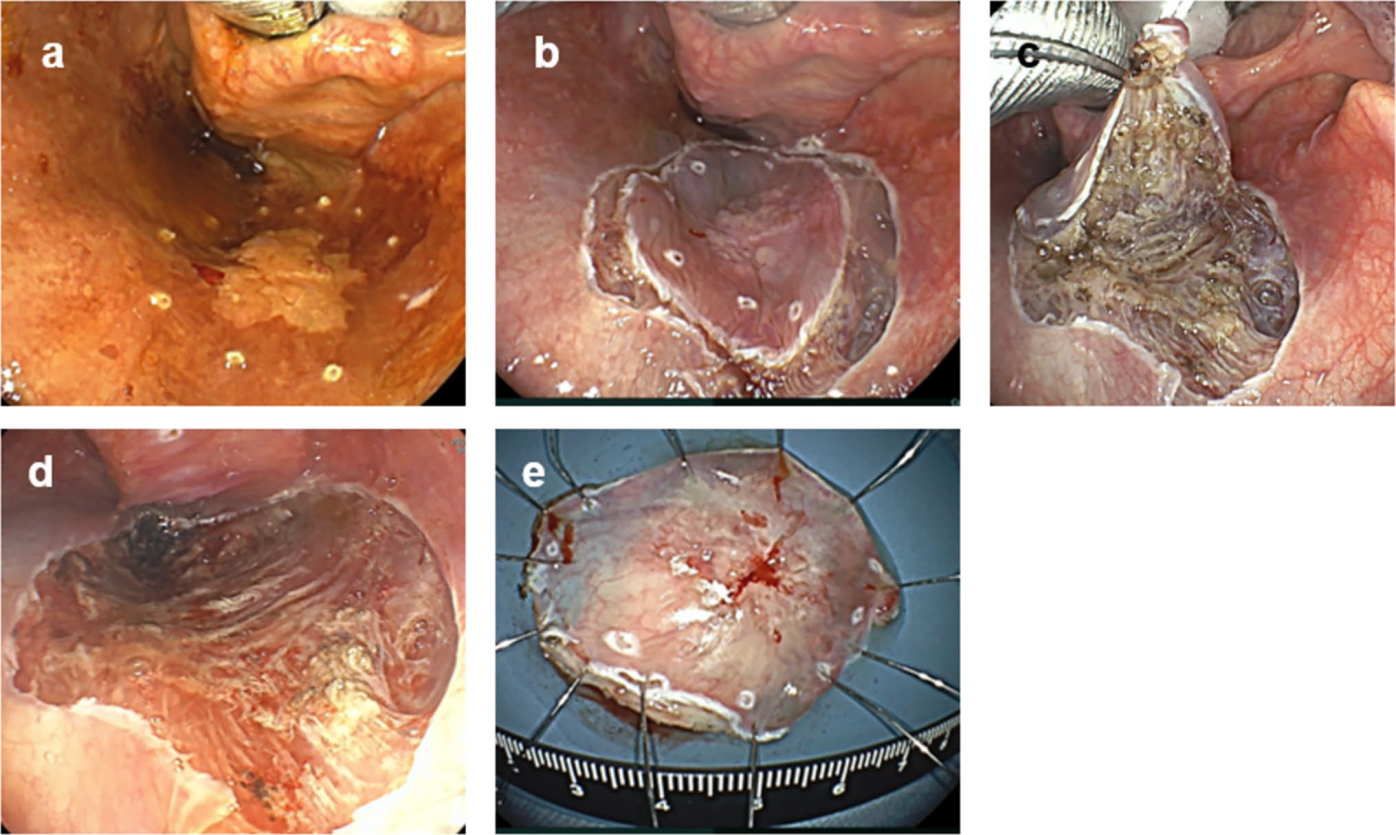
ELPS procedure. **a** A 2–3 mm margin is placed around the tumor. **b** Mucosal incision is made outside the margin. **c** Saline solution with indigocarmine is injected into the subepithelial propria and removed from the muscularis propria using an electrocautery. **d** After removal of the tumor. **e** Extraction specimen

Satou et al. developed the ELPS technique using a curved laryngoscope (Nagashima Medical Instruments Co., Ltd., Tokyo, Japan) to expand the hypopharynx (Fig 9), a high-resolution soft endoscope that provides an excellent surgical field, saline injection into the subepithelial layers to make the layers to be resected easier to recognize, and a grasping forceps and electrocautery to perform the operation [34] (Fig10). Watanabe et al. investigated the outcomes in 258 patients treated by ELPS and reported a 3-year OS of 85.7%. The curved laryngoscope facilitates development of the operative field and is currently also used for EMR and ESD. EMR, ESD, and ELPS using a curved laryngoscope are all less invasive as compared with TLM and TOVS, because injection of saline or other solution into the subepithelial layer in this operative technique minimizes invasion of the fascia, blood vessels, and nerves deep within the tumor [35]. While ELPS offers a good field of view and the surgeon can use both hands, mastering use of the curved device requires skill and can be technically challenging.

As mentioned above, there are several surgical options for TOS, and appropriate selection of the treatment method (especially endoscopic resection method) is important (Table 1). In esophageal cancer, there are limits to the size of tumors that can be resected *en bloc* by EMR [36], and segmental resection is the strongest prognostic factor for local recurrence [37]. Similarly, EMR is acceptable for SLPC if the lesion is small or can be adequately lifted by injection, but ESD is considered better in other cases. ESD is undoubtedly technically more challenging, however, and it has been shown that incomplete resection may be the cause of local recurrence, making it important to select the appropriate technique [38]. Although vertical margins have not been studied in detail, positive vertical margins after endoscopic treatment of esophageal cancer have been found to be a risk factor for recurrence [39], and additional treatment is recommended, which is an issue that needs further study. As the treatment strategy for SLPC, a “resect and watch” strategy has been proposed, in which patients are followed up after resection until the development of local recurrence, cervical lymph node metastasis, or metachronous pharyngeal carcinoma, without radical resection or additional radiation therapy, even if incomplete resection is achieved. However, close observation is recommended for patients with positive vertical margins, so as to avoid overlooking recurrence [40, 41].

**Table 1:** Characteristics of each treatement method TOS

	Indication	Instrument	Advantage	Disadvantage
TLM	oral, oropharynx, glottis, supraglottis, hypopharynx	Direct laryngoscope, CO2 laser	Accurate incisions and hemostasis using a microscope	Narrow field of view, Segmented resection
TOVS	oropharynx, supraglottis, hypopharynx	FK laryngopharyngoscope, straight devices	Straight devises, Tactile, Both hands can be used	Difficulty in developing the surgical field of the hypopharynx
EMR	oropharynx, supraglottis, hypopharynx, cervical esophagus	Gastrointestinal endoscopy, curved laryngoscope	Short treatment time	Larger lesions will be resected in segments
ESD	oropharynx, supraglottis, hypopharynx, cervical esophagus	Gastrointestinal endoscopy, curved laryngoscope	Visible line of resection, En-bloc resection is possible	Difficult to operate endoscope
ELPS	oropharynx, supraglottis, hypopharynx	Gastrointestinal endoscopy, curved laryngoscope, curved devices	Good surgical field, Both hands can be used	Difficult to operate curved devices

## Complications of TOS

TOS, including ELPS, has been reported as a minimally invasive, safe, and effective treatment for SLPC, and although a certain proportion of the patients develop complications, most complications are transient [33, 35, 42].

1. Postoperative bleeding.

The reported postoperative bleeding rate in patients undergoing TOS is around 5% [43, 44], and as in any surgery, use of antithrombotic medications is considered as a risk factor for postoperative bleeding [45–47]. The most common site of bleeding is from the postcricoid region of the hypopharynx, which contains a branch of the superior thyroid artery running through it and should be carefully monitored [44].

2. Laryngeal edema.

Laryngeal edema is considered as the most frequent complication of TOS [48] and is caused by extensive resection in the arytenoid and postcricoid regions, prolonged surgery, and increased local injection volume. Considering the possibility of laryngeal edema, in addition to steroid administration, prolonged tracheal intubation or tracheostomy until the following day should be considered in some cases [49, 50].

3. Perforation (Subcutaneous Emphysema, Mediastinitis, Mediastinal Abscess).

Involvement of the muscular layer may cause subcutaneous emphysema. In the posterior wall of the hypopharynx, excision of the prevertebral muscles may result in mediastinitis or mediastinal abscess [51, 52].

4. Stenosis, Dysphagia.

Postoperative dysphagia is usually transient, but often results in aspiration pneumonia. Older age (> 65 years), pulmonary dysfunction, and adhesions and deformities of the hypopharynx due to arytenoid resection and large mucosal defects have also been reported as risk factors for aspiration pneumonia [43, 45, 52]. There are no established methods for preventing adhesions in the hypopharynx, although the Valsalva maneuver and local steroid injections, which are used in esophageal cancer treatment, are reported as being useful [53].

## Prognosis

In general, patients with SLPC have a favorable prognosis with appropriate treatment, but in some cases, lymph node metastasis and multiple metachronous cancers may occur, which may exert an adverse effect on the prognosis [14, 45, 54, 55].

According to previous reports, the incidence of postoperative lymph node metastasis in SLPC is in the range of 1.9%–10% [14, 35, 44, 49]. In the case of superficial esophageal cancer, meta-analyses have reported that the tumor size, macroscopic tumor type, degree of tumor differentiation, depth of tumor invasion, and lymphatic or venous invasion are risk factors for lymph node metastasis [56]. However, since the head and neck region lacks a muscularis mucosa, it is difficult to evaluate the depth of the disease in a stratified manner. Therefore, the depth of SLPC is evaluated using tumor thickness as an indicator. Okabe et al. reported the following as possible risk factors for lymph node metastasis in patients with SLPC: type 0–IIa lesion on macroscopic examination, B2/B3 vessels on NBI, pathologic T stage ≥ T2, lymphatic/venous invasion, positive resection margins, and tumor thickness > 1000 μm [57]. However, while some reports showed a positive correlation between the tumor size and thickness, the variation was large, and the TNM classification in the current form is not sufficient to identify high-risk groups for lymph node metastasis based on the tumor thickness, and further accumulation of data is needed [58, 59]. Kitajima et al. studied the cutoff value of tumor thickness for the risk of lymph node metastasis, citing that in nonpedunculated submucosal invasive colorectal cancer, submucosal invasion of less than 1000 μm is associated with a 0% rate of lymph node metastasis [60]. They reported that the tumor thickness is a more sensitive indicator than the presence of lymphatic/venous invasion for predicting metastasis. Many subsequent reports have also reported a tumor thickness of > 1000 mm and presence of lymphatic/venous invasion as significant risk factors for lymph node metastasis [61, 62]. Budding has also been reported as another risk factor [63–65]. Budding is an isolated single cancer cell or a cluster of no more than 5 cancer cells, and in colorectal cancer, it is considered as a poor prognostic factor for OS [66]. Cancer-associated fibroblasts (CAFs) are believed to contribute to budding [67], and fibroblast activation protein (FAP)-positive CAFs have been reported as a risk factor for lymph node metastasis in esophageal cancer [68]. However, the role of CAFs in SLPC remains unclear, and further studies are needed.

Multiple primary cancers are known to occur at a high rate in patients with head and neck squamous cell carcinoma [69, 70]. This has been reported as field cancerization [71] and is an important concept to understand in today’s cancer practice. The rate of multiple primary cancers in patients with SLPC is higher than that reported above, and is around 30% [33, 43, 61]. This increase may be attributable to advances in diagnostic endoscopic technologies, which have facilitated the diagnosis of SLPC. It should be noted that more than 25% of patients with SLPC may have multiple primary cancers. Furthermore, metachronous cancers have been reported in nearly 20% of patients with SLPC [13, 72]. Evaluation of the number of Lugol-voiding lesions has been reported as being useful for predicting metachronous carcinoma [73], and in turn, the frequency of *TP53* mutations has been reported to be associated with the number of Lugol-voiding lesions. This may result from the accumulation of acetaldehyde, suggesting the need for patients to abstain from alcohol to reduce the risk of metachronous carcinogenesis, as well as the need for close surveillance [74, 75].

## Conclusion

In this review, we have outlined the epidemiology, general characteristics, diagnosis, treatment, and prognosis of SLPC. SLPC remains a relatively new disease concept that requires further validation. We believe that it is essential to continue accumulating evidence, especially in regard to evaluation of the tumor thickness, which cannot be addressed by the current TNM classification, and the risk factors for lymph node metastasis.
